# Cognitively normal Hispanic/Latino women demonstrate longitudinal learning and memory performance robust to inflammatory marker TNF‐α

**DOI:** 10.1002/alz70857_103891

**Published:** 2025-12-25

**Authors:** Taylor F Levine, Genna M Mashinchi, Emily Post, Catherine Chiao, Katie Stypulkowski, Nikki L Kaplan, Jessica ZK Caldwell

**Affiliations:** ^1^ Cleveland Clinic Lou Ruvo Center for Brain Health, Las Vegas, NV, USA; ^2^ Cleveland Clinic, Las Vegas, NV, USA; ^3^ Nevada Exploratory Alzheimer's Disease Research Center, Las Vegas, NV, USA

## Abstract

**Background:**

Women demonstrate an initial verbal learning and memory advantage at the earliest stages of Alzheimer's disease (AD) but more rapid cognitive decline following diagnosis. The mechanisms underlying these sex differences are not well understood. Inflammation represents one potential contributing factor, as inflammatory responses differ by sex and contribute to accumulation of AD pathology. Work from our group in a predominately non‐Hispanic white (NHW) cohort suggests that women demonstrate longitudinal verbal learning and memory performance robust to low‐to‐moderate levels of inflammatory markers but not high levels of inflammatory burden (Caldwell et al., 2021). Here, we investigated inflammation‐based sex differences in an ethnically diverse AD cohort.

**Method:**

We obtained data from the Health and Aging Brain Study‐Health Disparities and included 661 self‐reported NHW and 631 Hispanic/Latino (HL) participants who completed baseline and follow‐up testing (18‐24 months post baseline). We examined longitudinal sex differences in impact of inflammation (i.e., plasma tumor necrosis factor alpha (TNF‐α), interleukin 6 (IL‐6), and interleukin 10 (IL‐10)) on verbal learning and memory (Spanish English Verbal Learning Test learning (sum of trials 1‐5) and delayed recall). We hypothesized that cognitively normal (CN; CDR=0) women would show preserved learning and memory in the presence of increased inflammatory markers, but mildly cognitively impaired (MCI; CDR = .5) women would not.

**Result:**

A significant moderation effect of sex was observed for CN HL individuals, such that women retained verbal learning and memory regardless of TNF‐α levels, whereas CN men showed greater decline in verbal learning and memory with increased TNF‐α (*p* = .035 and *p* = .062, respectively). For MCI HL individuals, sex did not moderate the relationship between verbal learning and memory and TNF‐α. In the NHW cohort, sex did not moderate the relationship between inflammatory markers and verbal learning and memory in the CN and MCI groups. All results remained unchanged when controlling for number of anti‐inflammatory medications prescribed.

**Conclusion:**

Female‐specific verbal learning and memory advantages varied across ethnic groups in the presence of inflammatory marker TNF‐α. Further work is needed to parse complex patterns of resilience and vulnerability across racial and ethnic groups, and to understand causal factors underlying these patterns.

